# Bulbar function in children with spinal muscular atrophy type 1 treated with nusinersen

**DOI:** 10.1111/dmcn.16387

**Published:** 2025-06-12

**Authors:** Georgia Stimpson, Lavinia Fanelli, Eleanor Conway, Emily Johnson, Beatrice Berti, Mariacristina Scoto, Francesco Muntoni, Eugenio Mercuri, Giovanni Baranello, the p‐FOIS/CEDAS and OrSAT Working Group, Nikki Cornell, Giulia Stanca, Giorgia Coratti, Marika Pane

**Affiliations:** ^1^ Dubowitz Neuromuscular Centre UCL Great Ormond Street Institute of Child Health London UK; ^2^ Pediatric Neurology Unit Catholic University Rome Italy; ^3^ Centro Clinico NeMO Fondazione Policlinico Universitario Agostino Gemelli Istituto di Ricovero e Cura a Carattere Scientifico Rome Italy; ^4^ National Institute for Health and Care Research Great Ormond Street Hospital Biomedical Research Centre London UK; ^5^ Speech and Language Therapy Department Great Ormond Street Hospital London UK; ^6^ Neuromuscular Department Great Ormond Street Hospital London UK

## Abstract

**Aim:**

To describe bulbar function trajectories in patients with spinal muscular atrophy (SMA) type 1 treated with nusinersen in the UK and Italy.

**Method:**

In two previously reported, retrospective, observational cohort studies, we observed the 2‐year change in the Children's Eating and Drinking Ability Scale (CEDAS) (the revised and optimized version of the Paediatric Functional Oral Intake Scale [p‐FOIS]) and Oral and Swallowing Ability Tool (OrSAT) in 44 patients treated on average at 8.3 months (interquartile range = 4.1–14.4 months), with data collected every 6 months from treatment initiation. [Correction added on 30 June 2025 after first online publication: In the preceding sentence, 11.2 months (interquartile range = 4.1–24.7 months) has been changed to 8.3 months (interquartile range = 4.1–14.4 months).]

**Results:**

The Italian cohort had more participants in the 1b group (symptom onset >2 weeks and <3 months), while the UK had more participants in the 1c group (symptom onset <6 months). Over 2 years, the p‐FOIS/CEDAS captured lack of bulbar improvement in the 1b group, with 40% displaying stability and 45% showing decline; in the 1c group, stability was captured (71%). OrSAT captured improvement in 47% of the 1b group and 43% of the 1c group at 2 years; this was predominantly because of age‐related speech acquisition and feeding viscosities, where the item was not age‐appropriate at baseline.

**Interpretation:**

The p‐FOIS/CEDAS and OrSAT measures capture complementary information on the effect of disease‐modifying treatments (DMTs) on bulbar function. Further studies are required to understand bulbar function trajectories in symptomatic and presymptomatic cohorts with SMA receiving different DMTs.

## Graphical abstract

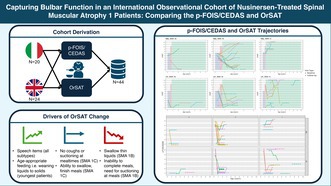

**Abbreviations**
CEDAS: Children's Eating and Drinking Ability Scale
DMT: disease‐modifying treatment
OrSAT: Oral and Swallowing Ability Tool
p‐FOIS: Paediatric Functional Oral Intake Scale
SMA: spinal muscular atrophy

**What this paper adds**

- The Paediatric Functional Oral Intake Scale/Children's Eating and Drinking Ability Scale and Oral and Swallowing Ability Tool (OrSAT) capture complementary aspects of bulbar function.
- Speech acquisition is the driver of OrSAT bulbar gains in spinal muscular atrophy type 1 (SMA1).
- Stable or declining bulbar function was evident after nusinersen treatment in individuals with SMA1.

Spinal muscular atrophy (SMA) is an autosomal recessive disease characterized primarily by progressive motor function decline, and variable bulbar and respiratory function. It is caused by mutations in the survival of motor neuron 1, telomeric (*SMN1*) gene, whose paralog, the *SMN2* gene, produces approximately 10% of the functional SMN protein. Classically, SMA is classified into three types, with patients with the most severe SMA (type 1) having a disease onset before 6 months and never sitting independently if untreated.^1^ SMA1 can be further classified into three subtypes: type 1a, which is characterized by neonatal symptom onset; type 1b, which is characterized by symptom onset before 3 months; and type 1c, which is characterized by symptom onset between 3 months and 6 months.^2^

The cohort with SMA1 is characterized by a significant bulbar function comorbidity, which manifests as feeding difficulties because of limited mouth opening, and facial, oral, and swallowing muscle weakness leading to insufficient food intake and aspiration of food and fluids. In natural history studies, most children with SMA1 require tube feeding by the age of 10 months.^3^, ^4^, ^5^, ^6^

Approval of three disease‐modifying treatments (DMTs) has drastically changed the phenotype of children with SMA, in particular those with SMA1, increasing survival and enabling the acquisition of unprecedented motor milestones. Nusinersen and risdiplam affect the alternative splicing of exon 7 of the *SMN2* gene, while onasemnogene abeparvovec is a gene therapy that replaces a patient's *SMN1* gene. However, data on the effects of DMTs on other aspects, including bulbar and respiratory function, are still limited. Additionally, currently there are no standardized measures for capturing bulbar function in paediatric patients; most adult measures of bulbar function are either inappropriate or not possible to administer in paediatric patients.

Both teams involved in the present study independently investigated the effects of nusinersen on bulbar function in treated SMA1 because this was the first DMT to become widely available to patients. Two scales were used separately, that is, the Paediatric Functional Oral Intake Scale (p‐FOIS), which is now known as the Children's Eating and Drinking Ability Scale (CEDAS),^7^ and the Oral and Swallowing Ability Tool (OrSAT). The p‐FOIS is a functional score related to feeding support, while the OrSAT is a structured checklist that captures bulbar impairment more broadly.

The study of the UK cohort showed that, despite improvements in motor function, bulbar function showed a marked decline when assessed using the p‐FOIS, with increased feeding support required in most patients, except for the oldest patients in the 1c group, who still had preserved bulbar function when starting treatment.^8^ In the Italian cohort, participants without a severe impairment at baseline, and who did not require tube feeding at baseline, displayed a broadly positive trend, although the caveat is that the maximum OrSAT score increases with age. Only participants with a ‘severe impairment’ on the OrSAT and a tracheostomy at baseline displayed a universal decline, similar to participants with a ‘severe impairment’ without a tracheostomy at baseline, although three participants in the latter group displayed an upward trajectory over time.^9^

The aim of this study was to combine and contrast the two cohorts to more fully understand bulbar function trajectories in treated children with SMA1. Additionally, we sought to understand what differentiates the oral and swallowing ability (OrSAT) of individuals based on their functional oral intake (p‐FOIS/CEDAS) at baseline. We also performed a shift analysis to understand which items of the OrSAT can drive possible changes in the whole cohort.

## METHOD

The observational data for the UK cohort were collected at Great Ormond Street Hospital between 2017 and 2020, while the data from the Italian cohort were collected at the Fondazione Policlinico Gemelli between 2017 and 2023. All participants included had SMA1 and were treated with nusinersen either soon after diagnosis or as soon as the drug became available to patients in the respective countries. In the UK cohort, the p‐FOIS data were already available from previous work and participants were scored retrospectively with the OrSAT using chart notes; similarly, the Italian cohort was scored retrospectively with the p‐FOIS, while OrSAT scores were already available from the previous study.^8^, ^10^ Participants were classified into SMA types 1a, 1b, and 1c based on age at symptom onset and clinical phenotype.

### Research ethics

This study was conducted in accordance with the Declaration of Helsinki. Each of the International SMA Consortium natural history studies had ethical approval in place that permitted the collection of SMA natural history data: SMA REACH UK: National REC London Bromley, Health Research Authority REC (reference no. 13/LO/1748, IRAS project ID: 122521). The Italian SMA Network is coordinated by the Catholic University of Sacred Heart, Fondazione Policlinico Universitario Agostino Gemelli Istituto di Ricovero e Cura a Carattere Scientifico (IRCCS) (institutional review board protocol no. 2533/18) and includes the University of Messina; the IRCCS Bambino Gesù Children's Hospital, Rome; the University of Milan, Niguarda Hospital; and IRCCS Istituto Giannina Gaslini, Genoa. All participants gave written informed consent to participate in the site‐specific natural history studies.

### Outcomes

The p‐FOIS is a six‐level functional score, with a lower score indicating a greater need for bulbar support. Those with a score of 6 have an age‐appropriate oral intake, while those with a p‐FOIS score of less than 4 require tube feeding to meet some or all of their nutritional needs.^8^ The p‐FOIS was recently renamed CEDAS; additional details have been provided to assign a score without altering the construct of the scales and the functional levels. Consequently, we use the term p‐FOIS/CEDAS to facilitate cross‐referencing with previous work using the p‐FOIS/CEDAS.

OrSAT is a 12‐item structured checklist, with each item scored as a yes (1) or no (0); a higher score is indicative of stronger bulbar function.^10^ The OrSAT items include the ability to swallow across several viscosities, impairment at meal times, and language ability. The full scoring system is provided in Table 2  of Berti et al.^10^ The total score on the OrSAT changes with age: below 6 months, items 1 and 5 to 10 are assessed for a maximum score of 7; between 6 months and 9 months, items 2, 3, and 11 (swallowing semi‐liquids and semi‐solids, speaking one or more syllables) are assessed for a maximum score of 10; and above 10 months, items 4 and 12 (swallowing of solids, speaking one or more words) are assessed for a maximum score of 12. The OrSAT levels assess functional impairment: ‘none’ represents safe and efficient swallowing for all consistencies; ‘mild’ represents safe swallowing but the individual requires compensatory strategies or other interventions; ‘moderate’ represents the ability to swallow some food consistencies safely but also the need for oral supplements or tube feeding; and ‘severe’ represents the inability to swallow by mouth and the need for tube feeding. An OrSAT assessment was considered valid only when all age‐appropriate items had been scored. Consequently, any items scored before age‐appropriateness (± 1 week) were classified as not appropriate.

### Statistical analyses

Patients were scored at baseline, and then at 6, 12, 18, and 24 months after nusinersen treatment. When considering the change in p‐FOIS/CEDAS, OrSAT level, and OrSAT score, the 24‐month change from baseline was used. Significant differences at baseline were assessed using the Wilcoxon rank‐sum (for age and OrSAT at baseline) and Fisher's exact (for the OrSAT and p‐FOIS/CEDAS levels) tests. Agreement of change across the OrSAT and p‐FOIS/CEDAS was assessed using Cohen's kappa and linear weights. Patterns of change in OrSAT items (i.e. shift) for the 2‐year follow‐up period were also presented. Additionally, patterns in the OrSAT items at baseline were identified using hierarchical clustering.

## RESULTS

Information from 44 participants was collected, with complete OrSAT assessments performed in 35 (80%) participants at baseline, while the p‐FOIS/CEDAS was available for all participants at baseline. Overall, the median age at treatment was 8.3 months (interquartile range = 4.1–14.4 months). [Correction added on 30 June 2025 after first online publication: In the preceding sentence, 11.2 months (interquartile range = 4.1–24.7 months) has been changed to 8.3 months (interquartile range = 4.1–14.4 months).] Patient characteristics and baseline functional outcomes are summarized in Table 1. There was a significant difference in age when starting nusinersen between the Italian and UK cohorts (*p* = 0.022), with participants from the UK being older at baseline, but not when considering only specific SMA types (*p* = 0.4, *p* = 0.554, *p* = 0.102 for 1a, 1b, 1c respectively). However, the age range was much wider in the UK cohort treated with nusinersen in the 1a and 1c groups; lack of significance is probably because of small numbers.

**TABLE 1:** Baseline characteristics of the study cohort.

Country			SMA type		
		All	1a	1b	1c
Italy	Number of patients	20	3	12	5
	Number of OrSAT	17	3	10	4
	Age at treatment (months)	6 (4–8.8)	5.5 (3–5.8)	5.3 (4–6.4)	9.5 (8.5–11)
	OrSAT score	6 (4–7)	4 (2–5)	6 (4–7)	8 (7–9)
	OrSAT level	4 (1–4)	3 (1–4)	3 (1–4)	4 (4–4)
	p‐FOIS/CEDAS score	5 (4–5)	4 (2–4)	5 (4–5)	5 (5–5)
UK	Number of patients	24	3	9	12
	Number of OrSAT	18	3	5	10
	Age at treatment (months)	11.2 (4.1–24.7)	26.2 (13.6–38.6)	4 (3.6–10)	18.6 (10.3–32.5)
	OrSAT score	7 (2–10)	2 (1–2)	7 (3–7)	10 (6–12)
	OrSAT level	3 (1–4)	2 (1–2)	2 (1–3)	4 (1–4)
	p‐FOIS/CEDAS score	3 (1–6)	1 (1–2)	3 (1–5)	4 (2–6)

*Note*: Data are median (interquartile range) for continuous variables, unless stated otherwise.

Abbreviations: CEDAS, Children's Eating and Drinking Ability Scale; OrSAT, Oral and Swallowing Ability Tool; p‐FOIS, Paediatric Functional Oral Intake Scale; SMA, spinal muscular atrophy.

At baseline, there was no significant difference in OrSAT score (*p* = 0.538 across all, and *p* = 0.506, *p* = 0.8, and *p* = 0.475 for SMA types 1a, 1b, 1c respectively). However, there was a significant difference in p‐FOIS/CEDAS across all groups (*p* < 0.001) and in the 1c group (*p* < 0.001), with participants in the UK cohort scoring on average worse than patients in the Italian cohort. The same trend was observed in the UK 1a and 1b groups compared to the Italian 1a and 1b groups, but this was not significant (*p* = 0.4, *p* = 0.286 respectively). There was no significant difference in OrSAT levels either across all groups (*p* = 0.165) or in SMA types 1a and 1c (*p* = 0.6, *p* = 0.914 respectively); however, in the 1b group (*p* = 0.005), the OrSAT level was on average lower in the UK versus the Italian cohort.

### Trajectories of the Paediatric Functional Oral Intake Scale and Oral and Swallowing Ability Tool score and level

**FIGURE 1:**
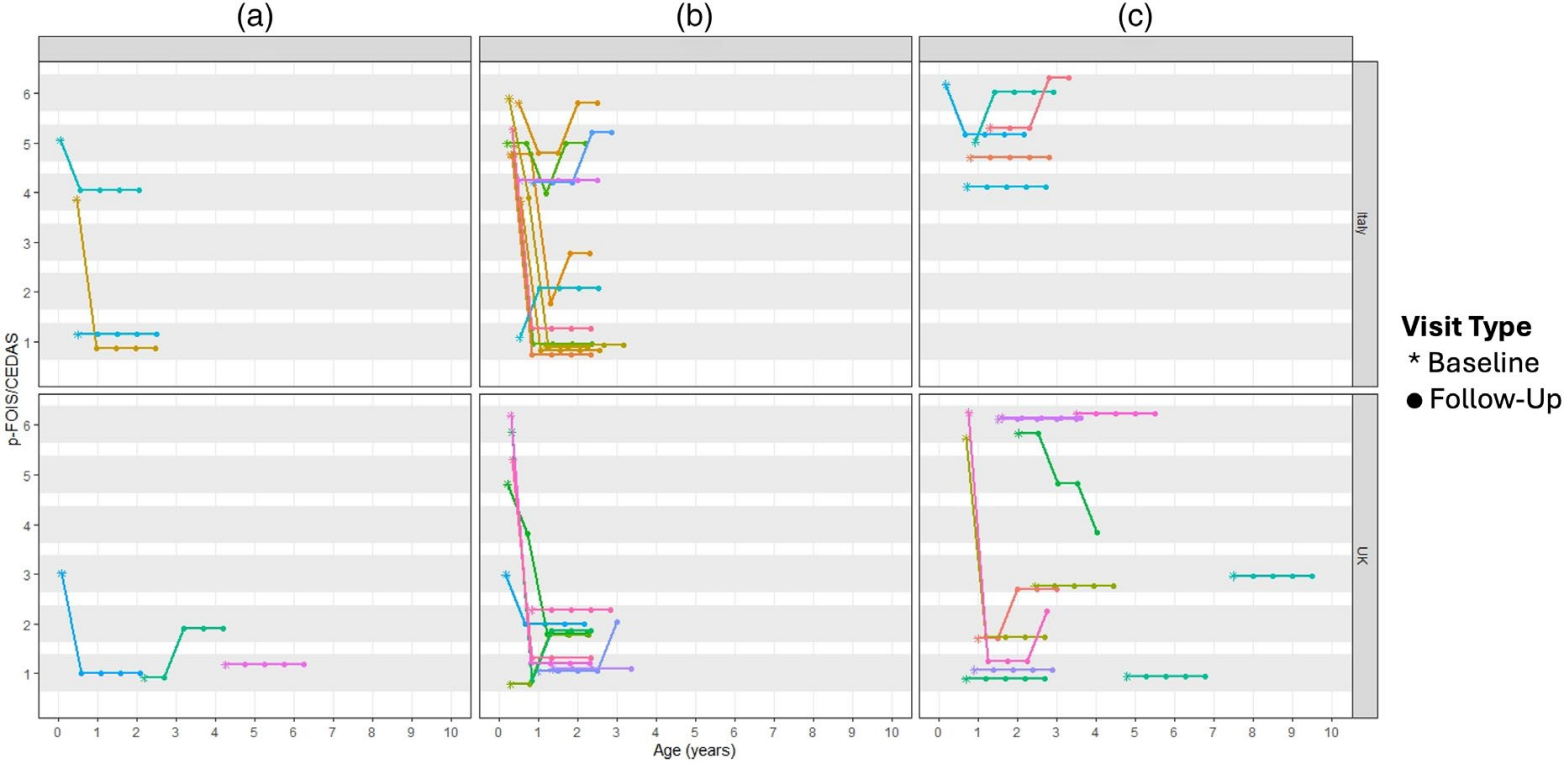
Paediatric Functional Oral Intake Scale (p‐FOIS)/Children's Eating and Drinking Ability Scale (CEDAS) trajectory according to age and country by SMA type (1a, 1b, 1c).

**FIGURE 2:**
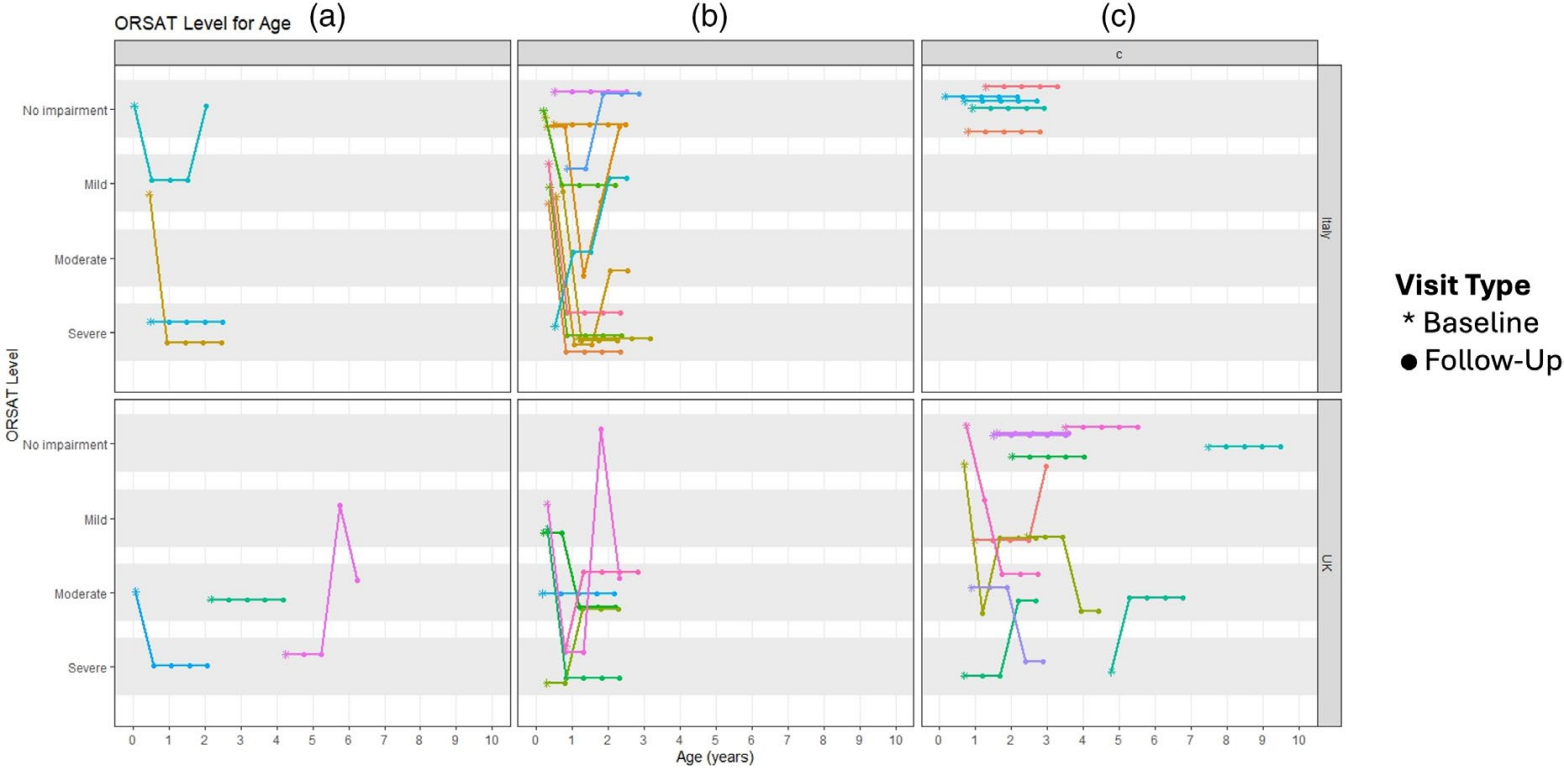
Oral and Swallowing Ability Tool (OrSAT) level trajectory according to age and country by SMA type (1a, 1b, 1c).

**FIGURE 3:**
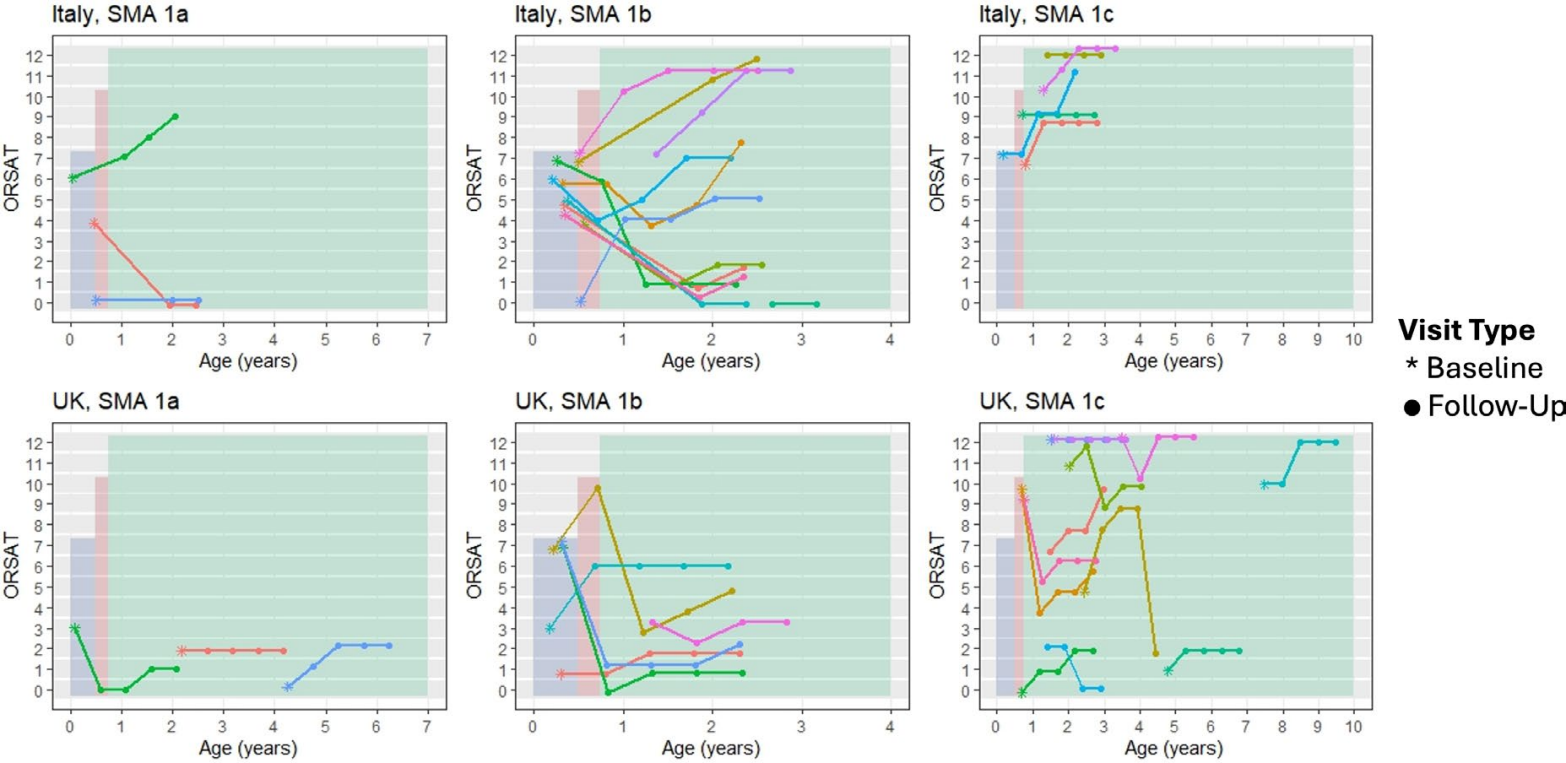
Oral and Swallowing Ability Tool (OrSAT) score trajectory according to age and country by SMA type (1a, 1b, 1c). Abbreviation: SMA, spinal muscular atrophy.

The trajectories of the p‐FOIS/CEDAS, and the OrSAT scores and levels, are shown in Figures 1, 2 to 3, with the overall trajectory summaries shown in Table 2.

**TABLE 2:** Summary of change in trajectory direction according to SMA type and outcome.

Count (percentage)		Outcome		
		p‐FOIS/CEDAS	OrSAT score	OrSAT level
All	Increased	6 (14)	15 (43)	5 (14)
	Stable	24 (56)	6 (17)	16 (46)
	Decreased	13 (30)	14 (40)	14 (40)
1a	Increased	1 (17)	2 (33)	1 (17)
	Stable	4 (67)	2 (33)	3 (50)
	Decreased	1 (17)	2 (33)	2 (33)
1b	Increased	3 (15)	7 (47)	2 (13)
	Stable	8 (40)	0 (0)	5 (33)
	Decreased	9 (45)	8 (53)	8 (53)
1c	Increased	2 (12)	6 (43)	2 (14)
	Stable	12 (71)	4 (29)	8 (57)
	Decreased	3 (18)	4 (29)	4 (29)

*Note*: Data are *n* (%).

Abbreviations: CEDAS, Children's Eating and Drinking Ability Scale; OrSAT, Oral and Swallowing Ability Tool; p‐FOIS, Paediatric Functional Oral Intake Scale; SMA, spinal muscular atrophy.

#### Paediatric Functional Oral Intake Scale/Children's Eating and Drinking Ability Scale scores

In the whole cohort, the p‐FOIS/CEDAS scores remained stable in 24 (55%), decreased in 13 (30%), and increased in six (14%) participants. In the 1a group, the p‐FOIS/CEDAS scores were stable in four (67%) participants. In the 1b group, nine (45%) participants showed a decline in p‐FOIS/CEDAS over time, while eight (40%) remained stable and three (15%) improved. Crucially, all nine participants who declined lost the ability to oral‐feed (p‐FOIS/CEDAS ≥ S3). In the Italian cohort, four participants in the 1b group maintained oral feeding after treatment; this was not observed in the UK cohort. In the 1c group, most participants were stable (12, 71%), with three participants declining (18%), and two improving (12%).

#### Oral and Swallowing Ability Tool levels

In the whole cohort with complete OrSAT assessments, OrSAT levels remained stable in 16 (46%), decreased in 14 (40%), and increased in five (14%). The 1a group showed some variability, with only one (17%) participant improving, three (50%) participants remaining stable, and two (33%) declining. In the 1b group, right (53%) participants displayed decline over 2 years, five (33%) remained stable, and two (13%) improved. In the 1c group, eight (57%) remained stable, four (29%) declined, and two (14%) improved, with all 1c participants in Italy showing no impairment at all time points.

#### Oral and Swallowing Ability Tool scores

In the whole cohort, the OrSAT scores remained stable in six participants (17%), decreased in 14 (40%), and increased in 15 (43%). The 1a group had variable trajectories (two stable, two improved, and two declined). The score showed an increasing trend after 2 years in seven participants (47%) from the 1b group, while eight (53%) participants showed a decline. In the 1c group, six participants (43%) improved, four (29%) were stable, and four (29%) declined.

### Shift of Oral and Swallowing Ability Tool items

The patient‐level item analysis is shown in Table 3; item‐level summaries are presented in Table S1. Items where the participant was unable to score at baseline because of age, but scored 1 at the follow‐up (referred to as age‐related acquisitions), drove the increasing scores. When these were removed, only three participants (50%) of the 1a group were either increasing or stable, two participants (13%) in the 1b group were increasing or stable, and five participants (36%) in the 1c group were increasing or stable.

**TABLE 3:** Twenty‐four‐month change in OrSAT items according to participant, stratified according to SMA subtype and age at treatment.

Subtype	Baseline score			Nusinersen Age (months)	24‐month change in item (colour) and baseline score (dots)												Change from baseline (full scores only)	
	OrSAT score	OrSAT level	p‐FOIS/CEDAS		1‐Swallow thin liquids	2‐Swallow semi‐liquids	3‐Swallow semi‐solids	4‐Swallow solids	5‐Need for intervention	6‐Cough during meals	7‐Swallow without tiring	8‐Able to complete meals	9‐Duration of meals	10‐Suctioning at meals	11‐Speak one syllable	12‐Speak one word	All items	Excluding age‐related acquisitions
1a	6	No impairment	5	0.5	○			◓	○	○				○			3	−1
	3	Moderate	3	1.0		◓	◓	◓	●		●	●	●			◓	−2	−3
	4	Mild	4	5.5		◓	◓	◓			●	●	●		◓	◓	−4	−4
	0	Severe	1	6.0	●	◓	◓	◓	●	●	●	●	●	●	◓	◓	0	0
	2	Moderate	1	26.2	●	●	●	●	●	●	●	●	●	●	○	○	0	0
	0	Severe	1	51.0	●	●	●	●	●	●	●	●	●	●			2	2
1b	3	Moderate	3	2.2		◓			●	○	●	●	●	○			3	−1
	6	No impairment	5	2.5	○			◓			○	○	●	○		◓	1	−2
	7	Mild	6	2.6		◓								○			−2	−6
	7	No impairment	1	3.1		◓	◓	◓								◓	−6	−7
	1	Severe	6	3.6	●	◓	◓	◓	●	●	●	●	●				1	−1
	7	Mild	5	3.7		◓	◓	◓									−5	−7
	6	No impairment	6	3.8	○							○					2	−3
	7	Mild	5	4.0		◓	◓	◓								◓	−6	−7
	5	Mild	5	4.1		◓	◓	◓			●		●				−3	−5
	4	Mild	5	4.2		◓	◓	◓		●	●		●			◓	−3	−4
	5	Mild	6	4.6		◓	◓	◓			●		●		◓	◓	−5	−5
	7	No impairment	4	6.0	○				○	○	○	○	○	○			5	0
	7	No impairment	1	6.1	○				○		○	○	○	○	○		4	−1
	0	Severe	4	6.4	●			◓	●	●	●	●	●				5	4
	4	Mild	2	6.7			●	◓	●	●	●		●				−2	−3
1c	7	No impairment	6	2.0	○				○	○	○	○		○			4	−1
	10	No impairment	6	8.3	○		○	◓		○				○	○		−4	−5
	0	Severe	1	8.3	●	●	●	◓	●	●	●	●	●	●			2	1
	9	No impairment	4	8.5	○	○		◓		○	○	○		○	○		0	−1
	9	No impairment	6	9.0		○	○	◓		○				○			−3	−4
	7	No impairment	5	9.5	○	○	○	◓	●	○		○	●	○	○		2	1
	10	No impairment	5	15.5	○	○	○	○	○	○	○	○		○	○		2	2
	12	No impairment	6	18.0	○	○	○	○	○	○	○	○	○	○	○	○	0	0
	12	No impairment	6	19.2	○	○	○	○	○	○	○	○	○	○	○	○	0	0
	11	No impairment	6	24.2	○	○	○			○		○	○	○	○	○	−1	−1
	5	Mild	3	29.3	●		●		●		●	●	●		○		−3	−3
	12	No impairment	6	42.0	○	○	○	○	○	○	○	○	○	○	○	○	0	0
	1	Severe	1	57.4	●	●	●	●	●	●	●	●	●	●	○		1	1
	10	No impairment	3	89.8	○	○	○	○	○	○	○			○	○	○	2	2

*Note*: Yellow is stable over time (1 to 1 [○] or 0 to 0 [●] or not appropriate to 0 [◓]); red is loss over time (1 to 0); green is gain over time (0 to 1); and blue is gain when previously not appropriate (not appropriate to 1).

Abbreviations: CEDAS, Children's Eating and Drinking Ability Scale; OrSAT, Oral and Swallowing Ability Tool; p‐FOIS, Paediatric Functional Oral Intake Scale; SMA, spinal muscular atrophy.

Of the participants who were able to swallow thin liquids at baseline (OrSAT item 1), 56% continued to do so at 24 months; 92% who lost this ability were either type 1a or 1b. For the other swallowing items, that is, 2 (semi‐liquids), 3 (semi‐solids), and 4 (solids), 12, nine, and seven participants respectively had this ability at baseline; of these, 25%, 0%, and 29% lost this ability by the 24‐month follow‐up. In participants where the item was not assessed at baseline because of age, 33%, 44%, and 24% were able to swallow semi‐liquids, semi‐solids, and solids respectively at the 24‐month follow‐up. Items 5 to 10 (‘need for intervention’, ‘cough/signs of stagnation during meal’, ‘able to swallow without tiring’, ‘able to complete a meal’, ‘duration of main meals’, and ‘need for suctioning during mealtime’), scored 63%, 74%, 51%, 66%, 43%, and 83% at baseline respectively. However, approximately half of participants who scored at baseline no longer scored on these items at the 24‐month follow‐up (57%, 46%, 42%, 44%, 56%, and 41% respectively), mostly participants in group 1a or 1b.

Most notably, items 11 and 12 (‘speak one or more syllables’ and ‘speak correctly one or more words’ respectively) were not age‐appropriate in most participants with SMA types 1a and 1b at baseline (76% and 90% respectively); however, most participants in these groups gained these abilities (81% and 60% respectively), with no loss observed on these items.

### Comparison of Oral and Swallowing Ability Tool levels and the Paediatric Functional Oral Intake Scale (Children's Eating and Drinking Ability Scale)

When the OrSAT scores were scaled according to the number of items that were age‐appropriate for a given child, the OrSAT score showed a correlation of 0.77 and 0.88 at baseline for the OrSAT level and p‐FOIS/CEDAS respectively, and a correlation of 0.91 and 0.9 across all assessments with the OrSAT level and p‐FOIS/CEDAS respectively. There was significant agreement regarding the change in OrSAT level and p‐FOIS/CEDAS at the 24‐month follow‐up (53% agreement, 95% confidence interval = 33–73%). An overview of these changes is presented in Table 4. No change was detected on the p‐FOIS/CEDAS in seven participants when a change in OrSAT level was detected. No change was detected in the OrSAT in eight participants when a change was detected on the p‐FOIS/CEDAS. If a decline was observed on both the OrSAT and p‐FOIS/CEDAS, the magnitude of the decline on the p‐FOIS/CEDAS was always larger than the decline on the OrSAT.

**TABLE 4:** Comparison of 24‐month change in p‐FOIS/CEDAS and OrSAT level (granular).

	Change in p‐FOIS/CEDAS						
Change in OrSAT level	−5	−4	−3	−2	−1	0	1
−3	1	0	0	0	0	0	0
−2	0	5	1	0	0	0	0
−1	1	1	2	1	0	3	0
0	0	0	0	2	3	10	3
1	0	0	0	0	0	4	3
2	0	0	0	0	0	0	1

*Note*: Yellow is stable over time; red is loss over time; and green is gain over time.

Abbreviations: CEDAS, Children's Eating and Drinking Ability Scale; OrSAT, Oral and Swallowing Ability Tool; p‐FOIS, Paediatric Functional Oral Intake Scale.

### What differentiates participants at baseline?

**FIGURE 4:**
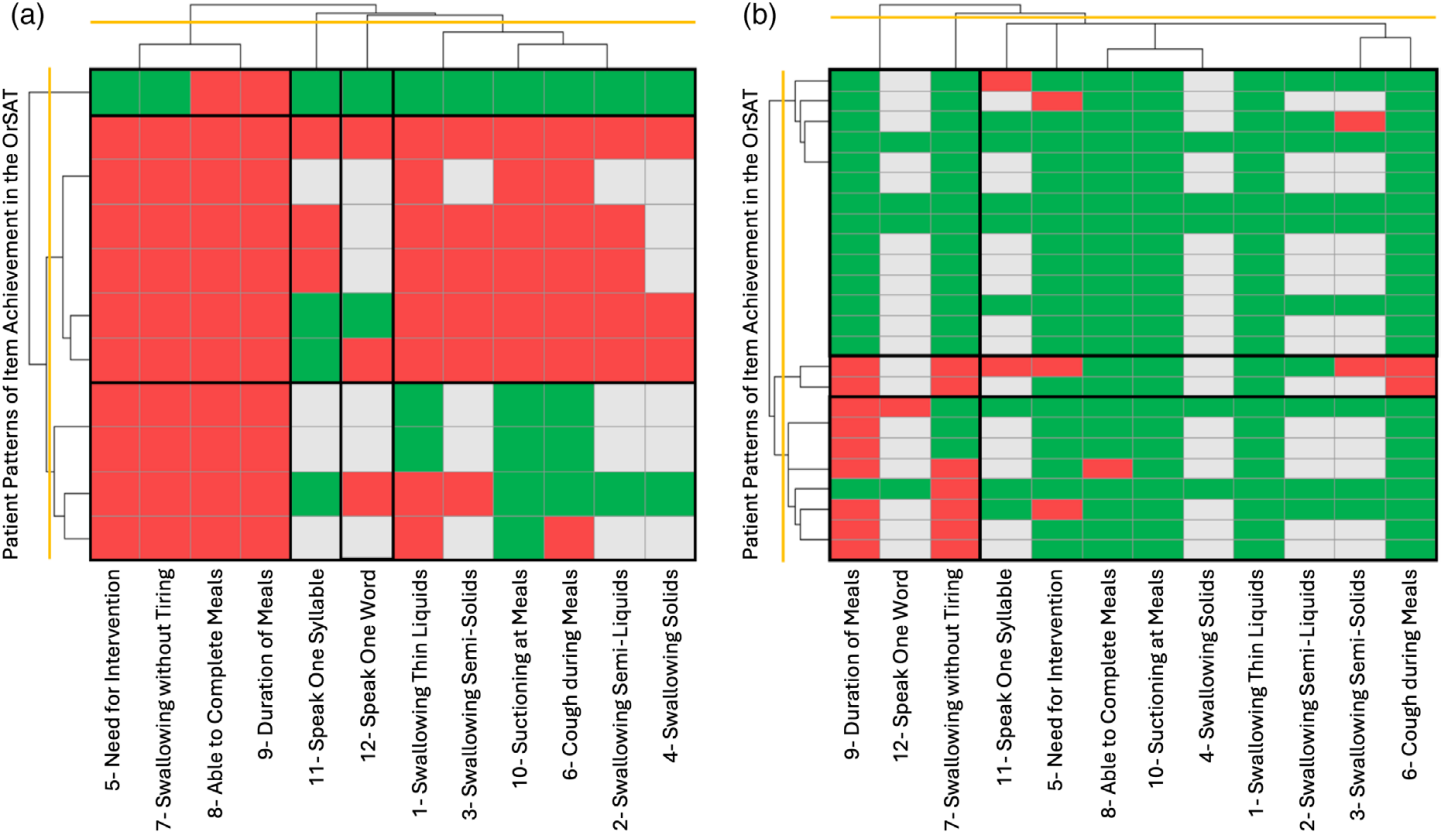
Patterns of Oral and Swallowing Ability Tool (OrSAT) achievement according to a Paediatric Functional Oral Intake Scale/Children's Eating and Drinking Ability Scale score ≤3 (a) and ≥4 (b).

In the 11 participants who were not eating orally at baseline (p‐FOIS/CEDAS ≤3), a co‐occurring inability on items 5, 7, 8, and 9 of the OrSAT (‘need for thickened food or positioning’, ‘easily tired while swallowing’, ‘unable to complete a meal’, and ‘long meal durations’ respectively) was evident in 91% of participants (Figure 4a). Additionally, six of these participants (55%) scored a 0 (were completely unable) across all of the age‐appropriate OrSAT items, excluding speech‐related items (items 11 and 12). Conversely, in the 24 participants whose baseline p‐FOIS/CEDAS was at least 4, achievement on all age‐appropriate items was present in 46% of participants, with sporadic missingness of only one item in three further participants (Figure 4b). In the other 10 participants, items 9 (duration of meals) and 7 (swallow without tiring) were most often scored as a 0 in 90% and 70% of participants respectively.

## DISCUSSION

Bulbar aspects are less well understood than motor and respiratory function in SMA, and assessing bulbar function has become increasingly critical given new DMTs. Even in pivotal DMT clinical trials, bulbar function was not evaluated systematically. This can be partly explained by the paucity of specific bulbar function tools for children with neuromuscular disorders. While videofluoroscopy and detailed speech therapy assessments remain the criterion standard, new clinical tools have recently been proposed.^11^

In our study, we combined and compared the use of two recently developed measures, the p‐FOIS/CEDAS and OrSAT, to capture changes in bulbar function in participants with SMA1 treated with nusinersen.

We found that the two scales were highly correlated and could be used in combination to increase the level of accuracy when describing bulbar function abilities in treated participants with SMA1. The p‐FOIS/CEDAS can provide more detailed information, and a greater number of specifications, than OrSAT levels; it is more accurate in identifying different levels in participants with gastrostomy with a floor effect on the OrSAT levels. On the other hand, the OrSAT checklist items can provide insights into fatigue in children who are not tube‐fed. The acquisition of age‐appropriate items on the OrSAT scores also allows clinicians to assess patient progression through feeding milestones.

By combining these scales, we identified those items of the OrSAT that change with treatment, depending on functional level at baseline. Most participants who were still orally fed at baseline (p‐FOIS/CEDAS ≥4) completely or nearly completely performed all age‐appropriate OrSAT items; items that capture fatigue during feeding and prolonged meal duration (items 7 and 9) showed the most variability in this stronger patient population. Conversely, most participants who were already tube‐fed at baseline (p‐FOIS/CEDAS ≤3) showed complete inability across all age‐appropriate OrSAT items, except for those related to speech, with some variability in items capturing the ability to swallow liquids, the presence of cough or stagnation, or the need for suctioning during meals (items 1, 6, and 10).

This study also allowed us to identify some differences in the two populations of patients who were studied, with significantly older age at the start of treatment and worse bulbar function (assessed using the p‐FOIS/CEDAS) in the UK cohort compared to the Italian cohort. However, the levels of the abilities assessed using the total OrSAT scores were not different between the two cohorts. As shown in a previous study,^8^ participants in the 1b group were more likely to show a deterioration of bulbar function over time compared to participants in the 1c group, despite ongoing treatment. However, a few participants in the 1b group in the Italian cohort had preserved oral feeding on the p‐FOIS/CEDAS; this was not observed in the UK cohort. Possible differences in care and approach to tube feeding between the two countries, as shown in previous studies,^5^ could potentially explain these findings and highlight the need to further investigate the effects of specific training to preserve and improve bulbar function in patients with SMA receiving new DMTs. In particular, both scales describe functional ability but do not include an objective assessment on the safety of these abilities. Consequently, for both the p‐FOIS/CEDAS and OrSAT, it is possible for an individual to score higher or lower than their baseline objective function. Cultural and individual differences can influence this, for example, one family may prioritize minimizing aspiration risk in decision‐making around tube feeding, while another may prioritize the quality of life linked to oral eating and drinking and so push for oral intake for as long as possible.

Overall, improvements on the OrSAT at 24 months were predominantly driven by developmental gains rather than items that were age‐appropriate at both the baseline and follow‐up. These age‐related acquisitions may be interpreted as a response to treatment when acquisition of age‐appropriate milestones is never observed in the natural history of the disease. The acquisition or maturation of age‐appropriate feeding skills, such as progress through the weaning stages captured by the OrSAT items, could represent potential aspects to target with a rehabilitation approach; this could become part of the standard of care for patients with SMA1 treated with DMT. The weaning stages describe the progression from a purely liquid diet from birth to 6 months, to semi‐liquid, semi‐solid, and then solid food as neurodevelopmental milestones and dental eruption enable the child to safely process varied textures.

Other aspects driving change, such as acquisition of speech, were less relevant because they provide only some high‐level information that should be explored further. More appropriate assessments of speech development that would also consider other domains, such as language development and cognitive function, should be considered for individuals with SMA1.

In conclusion, our study suggests that the two measures provide complementary information and capture possible bulbar function activities and domains that are more susceptible to change after DMT. An increasing number of infants are being treated with the other two drugs available (risdiplam and onasemnogene abeparvovec); thus, further studies will be needed to describe bulbar function trajectories in larger cohorts with SMA treated with different therapies.

## CONFLICTS OF INTEREST STATEMENT

BB reports personal fees from Biogen, Novartis, and Roche outside the submitted work. EC reports a specialist adviser role in SMA for Roche Pharmaceuticals. EJ reports a clinical evaluator role in clinical trials for Novartis and teaching/consultancy for Biogen and Roche. EM reports personal fees from AveXis, Roche, Biogen, PTC Therapeutics, Sarepta, Santhera Pharmaceuticals, and Scholar Rock, all outside the submitted work. FM reports participation to scientific advisory boards and teaching initiatives for Novartis, Biogen, and Roche; he is involved as an investigator in clinical trials for Novartis, Biogen, and Roche. Both UCL and Great Ormond Street Hospital receive funding from Biogen and Roche for the SMA REACH SMA registry. GB is the principal investigator of clinical trials by Pfizer, NS Pharma, and ReveraGen BioPharma; has received speaker or consulting fees from Sarepta, Pfizer, PTC Therapeutics, Biogen, Novartis Gene Therapies (AveXis), and Roche; and has worked as the principal investigator on SMA studies sponsored by Novartis Gene Therapies and Roche. GC reports consultant fees from Biogen, Roche, Novartis, and Solid. MS is principal investigator in clinical trials for Roche, Biogen, Dyne, and Italfarmaco. She has received consultancy honoraria for membership of scientific advisory boards and teaching initiatives for Roche, Biogen, and Novartis. She is the co‐principal investigator of the SMA REACH UK network, currently cofunded by Roche, Biogen, and Novartis. NC reports honoraria from Roche. G Stimpson, G Stance, MP, and LF report no conflicts of interest.

## Supporting information


**Table S1:** Item‐level summaries.

## Data Availability

Data available on request from the authors.
