# Supplementary material for: Bulbar function in children with spinal muscular atrophy type 1 treated with nusinersen
Source: Dev Med Child Neurol. 2025 Jun 12;67(12):1590–600. doi: 10.1111/dmcn.16387 (PMC12618960; doi:10.1111/dmcn.16387)
Supplement: Supplementary file 1 — Table S1: Item‐level summaries. [file DMCN-67-1590-s001.docx]

**10 Appendix**

| 1A | Item | Loss | Stable  0->0 ● | Stable  NA -> 0 ◓ | Stable  1->1 ○ | Gain  0->1 | Gain  NA->1 |
| --- | --- | --- | --- | --- | --- | --- | --- |
| ORSAT Items | 1-Swallow Thin Liquids | 2 (33%) | 3 (50%) | 0 (0%) | 1 (17%) | 0 (0%) | 0 (0%) |
|  | 2-Swallow Semi Liquids | 0 (0%) | 2 (33%) | 3 (50%) | 0 (0%) | 0 (0%) | 1 (17%) |
|  | 3-Swallow Semi Solids | 0 (0%) | 2 (33%) | 3 (50%) | 0 (0%) | 0 (0%) | 1 (17%) |
|  | 4-Swallow Solids | 0 (0%) | 2 (33%) | 4 (67%) | 0 (0%) | 0 (0%) | 0 (0%) |
|  | 5-Need for Intervention | 1 (17%) | 4 (67%) | 0 (0%) | 1 (17%) | 0 (0%) | 0 (0%) |
|  | 6-Cough during Meals | 2 (33%) | 3 (50%) | 0 (0%) | 1 (17%) | 0 (0%) | 0 (0%) |
|  | 7-Swallow without Tiring | 1 (17%) | 5 (83%) | 0 (0%) | 0 (0%) | 0 (0%) | 0 (0%) |
|  | 8-Able to Complete Meals | 1 (17%) | 5 (83%) | 0 (0%) | 0 (0%) | 0 (0%) | 0 (0%) |
|  | 9-Duration of Meals | 0 (0%) | 5 (83%) | 0 (0%) | 0 (0%) | 1 (17%) | 0 (0%) |
|  | 10-Suctioning at Meals | 2 (33%) | 3 (50%) | 0 (0%) | 1 (17%) | 0 (0%) | 0 (0%) |
|  | 11-Speak One Syllable | 0 (0%) | 0 (0%) | 2 (33%) | 1 (17%) | 1 (17%) | 2 (33%) |
|  | 12-Speak One Word | 0 (0%) | 0 (0%) | 3 (50%) | 1 (17%) | 1 (17%) | 1 (17%) |
| 1B | Category | Loss | Stable  0->0 ● | Stable  NA -> 0 ◓ | Stable  1->1 ○ | Gain  0->1 | Gain  NA->1 |
| ORSAT Items | 1-Swallow Thin Liquids | 9 (60%) | 2 (13%) | 0 (0%) | 4 (27%) | 0 (0%) | 0 (0%) |
|  | 2-Swallow Semi Liquids | 1 (7%) | 0 (0%) | 9 (60%) | 0 (0%) | 1 (7%) | 4 (27%) |
|  | 3-Swallow Semi Solids | 0 (0%) | 1 (7%) | 7 (47%) | 0 (0%) | 1 (7%) | 6 (40%) |
|  | 4-Swallow Solids | 0 (0%) | 0 (0%) | 10 (67%) | 0 (0%) | 0 (0%) | 5 (33%) |
|  | 5-Need for Intervention | 8 (53%) | 4 (27%) | 0 (0%) | 2 (13%) | 1 (7%) | 0 (0%) |
|  | 6-Cough during Meals | 9 (60%) | 4 (27%) | 0 (0%) | 2 (13%) | 0 (0%) | 0 (0%) |
|  | 7-Swallow without Tiring | 5 (33%) | 7 (47%) | 0 (0%) | 3 (20%) | 0 (0%) | 0 (0%) |
|  | 8-Able to Complete Meals | 8 (53%) | 3 (20%) | 0 (0%) | 4 (27%) | 0 (0%) | 0 (0%) |
|  | 9-Duration of Meals | 5 (33%) | 8 (53%) | 0 (0%) | 2 (13%) | 0 (0%) | 0 (0%) |
|  | 10-Suctioning at Meals | 9 (60%) | 0 (0%) | 0 (0%) | 5 (33%) | 1 (7%) | 0 (0%) |
|  | 11-Speak One Syllable | 0 (0%) | 0 (0%) | 1 (7%) | 1 (7%) | 2 (13%) | 11 (73%) |
|  | 12-Speak One Word | 0 (0%) | 0 (0%) | 5 (33%) | 0 (0%) | 0 (0%) | 10 (67%) |
| 1C | Category | Loss | Stable  0->0 ● | Stable  NA -> 0 ◓ | Stable  1->1 ○ | Gain  0->1 | Gain  NA->1 |
| ORSAT Items | 1-Swallow Thin Liquids | 1 (7%) | 3 (21%) | 0 (0%) | 10 (71%) | 0 (0%) | 0 (0%) |
|  | 2-Swallow Semi Liquids | 2 (14%) | 2 (14%) | 0 (0%) | 9 (64%) | 0 (0%) | 1 (7%) |
|  | 3-Swallow Semi Solids | 0 (0%) | 3 (21%) | 0 (0%) | 9 (64%) | 1 (7%) | 1 (7%) |
|  | 4-Swallow Solids | 2 (14%) | 1 (7%) | 5 (36%) | 5 (36%) | 0 (0%) | 1 (7%) |
|  | 5-Need for Intervention | 4 (29%) | 4 (29%) | 0 (0%) | 6 (43%) | 0 (0%) | 0 (0%) |
|  | 6-Cough during Meals | 1 (7%) | 2 (14%) | 0 (0%) | 11 (79%) | 0 (0%) | 0 (0%) |
|  | 7-Swallow without Tiring | 2 (14%) | 3 (21%) | 0 (0%) | 7 (50%) | 2 (14%) | 0 (0%) |
|  | 8-Able to Complete Meals | 2 (14%) | 3 (21%) | 0 (0%) | 8 (57%) | 1 (7%) | 0 (0%) |
|  | 9-Duration of Meals | 4 (29%) | 4 (29%) | 0 (0%) | 4 (29%) | 2 (14%) | 0 (0%) |
|  | 10-Suctioning at Meals | 1 (7%) | 2 (14%) | 0 (0%) | 11 (79%) | 0 (0%) | 0 (0%) |
|  | 11-Speak One Syllable | 0 (0%) | 0 (0%) | 0 (0%) | 11 (79%) | 2 (14%) | 1 (7%) |
|  | 12-Speak One Word | 0 (0%) | 0 (0%) | 0 (0%) | 5 (36%) | 3 (21%) | 6 (43%) |

Supplementary Table S1: 24 month change in ORSAT items by SMA Type. (Key: Yellow is stable over time (1->1 (○) or 0->0 (●) or not appropriate ->0 (◓), red is loss over time (1->0), green is gain over time (0->1), blue is gain when previously not appropriate (Not appropriate ->1))
